# Gastroparesis With the Initiation of Liraglutide: A Case Report

**DOI:** 10.7759/cureus.11735

**Published:** 2020-11-28

**Authors:** Sami Almustanyir, Habeeb Alhabeeb, Noara AlHusseini, Mohammed Al Thow

**Affiliations:** 1 Department of Internal Medicine, Ministry of Health, Riyadh, SAU; 2 Department of Internal Medicine, King Fahad Medical City, Riyadh, SAU; 3 Medicine, Alfaisal University, Riyadh, SAU; 4 Medicine, King Faisal University, Al-Ahsa, SAU

**Keywords:** gastroparesis, liraglutide, nausea, gastric suctioning, diabetes mellitus, case report

## Abstract

Gastroparesis is a syndrome that is manifested by gastrointestinal symptoms and delayed gastric emptying without evidence of mechanical obstruction. Herein, we present the case of an 18-year-old diabetic female patient who developed clinical features suggestive of gastroparesis after initial doses of liraglutide. Although rare, drug-induced gastroparesis should be contemplated in diabetic patients with a history of recent commencement of liraglutide, particularly at higher doses. Management of drug-induced gastroparesis is largely symptomatic.

## Introduction

Gastroparesis is a functional disorder characterized by delayed gastric emptying [1,2]. It is linked to an array of symptoms comprising upper abdominal pain, bloating, and satiety. These symptoms can be significantly morbid for patients, affecting their wellbeing quality and resulting in repeated work absences as well as increased hospital visits [3].

Gastroparesis is increasingly becoming a burden on the healthcare industry due to its rising prevalence, incidence, and hospitalization frequencies [2]. Jung and colleagues conducted an extensive study to assess the incidence and prevalence of gastroparesis. The authors detected that the age-adjusted prevalence per 1000 individuals was 9.6 and 37.8 for males and females, respectively [4].

There is a substantial overlay between the symptoms of gastroparesis and functional dyspepsia. Both conditions share the findings of post-meal fullness and epigastric pain. Nonetheless, weight loss and nausea/vomiting are more frequently seen in gastroparesis rather than in functional dyspepsia [2]. Besides functional dyspepsia, the symptoms of gastroparesis have similarities with other conditions, such as gastritis and peptic ulcer [3]. A distinction in diagnosis is not always useful and can prove to be arbitrary, as the symptoms are similar and can last for three to six months before patients seek medical attention [2].

Gastroparesis has different etiologies. The most reported ones comprise diabetes mellitus and post-surgical procedures. Idiopathic causes are reported in up to one-third of the cases. Notably, diabetes mellitus is the leading systemic disease implicated in patients with gastroparesis [3].

Herein, we present the case of an 18-year-old diabetic female patient who developed clinical features suggestive of gastroparesis after initial doses of liraglutide.

## Case presentation

An 18-year-old female patient presented to the emergency room complaining of vomiting and upper abdominal discomfort for the past six days. Her pain was progressive, dull-aching, and non-radiating in nature. The severity of pain was seven out of ten. She had no other gastrointestinal symptoms. The review of her systems was unremarkable. She had no history of surgeries or allergies to drugs or food. She denied the use of herbal medications, tobacco, and alcohol. She was not sexually active and did not have any exposure to animals. Past medical history revealed significance for a four-year history of uncontrolled type-two diabetes mellitus which was managed with insulin therapy. Recently, she was started on three mg of liraglutide for the management of her diabetes mellitus and obesity. Family history was notable for diabetes mellitus, hypertension, and obesity.

On physical examination, she was found to be vitally stable. Her body mass index was 31.9 kg/m^2^. Her lungs were clear to auscultation with normal heart sounds. Abdominal exam displayed a distended abdomen with epigastric tenderness. 

Laboratory results revealed a blood glucose level of 170 mg/dL (normal range: less than 100 mg/dL). Her glycated hemoglobin A1C was 8.4% (normal: less than 6.5%). Her blood test results for creatinine, potassium, sodium, aspartate aminotransferase, and alanine aminotransferase were within normal levels.

**Figure 1:**
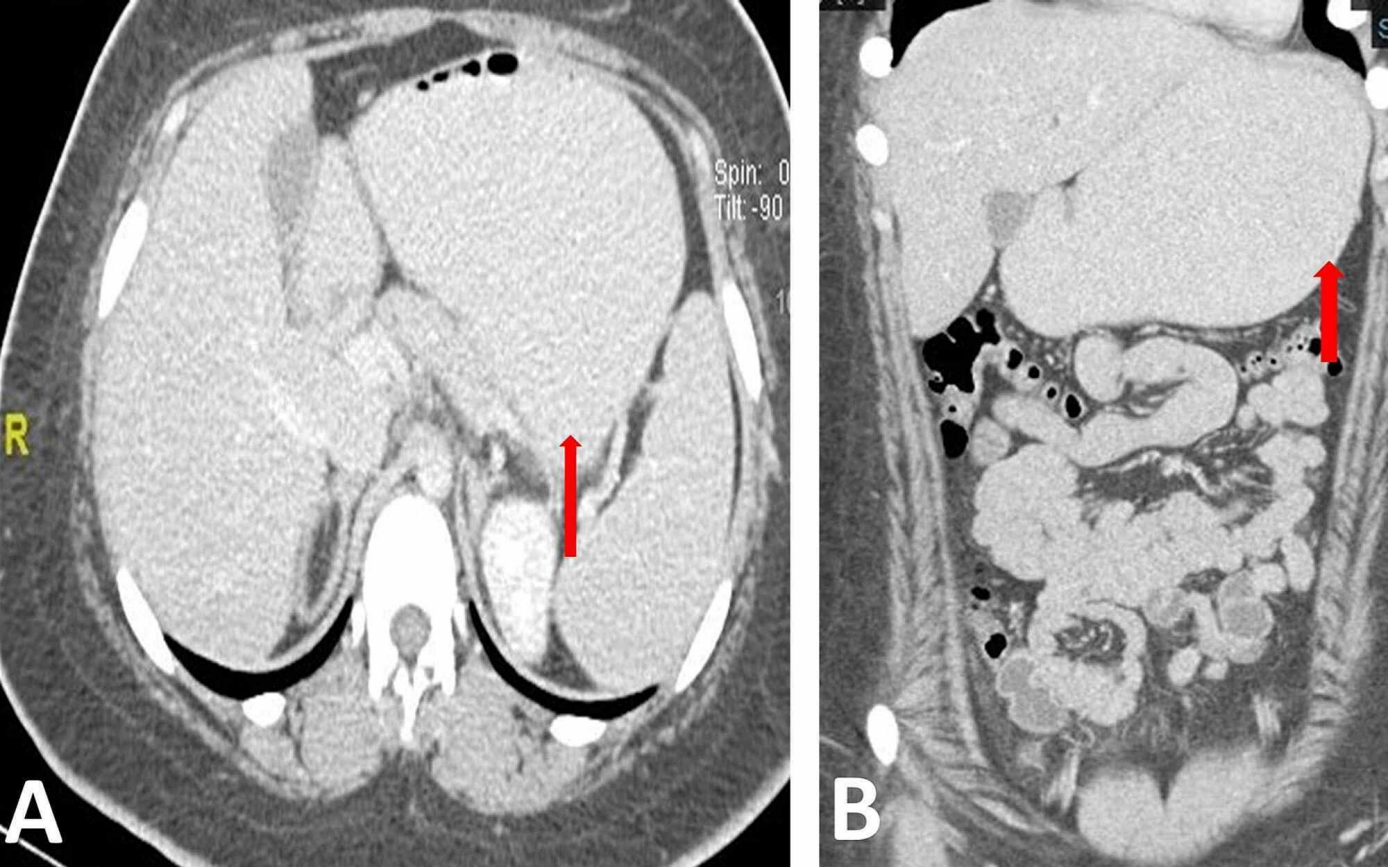
Sagittal (A) and coronal (B) computed tomography scans showing an isolated moderate-grade gastric distension (red arrow), without evidence of distal gastric, pyloric, or proximal duodenal obstructing masses.

X-ray of abdomen displayed a non-obstructive gas pattern without intestinal dilatation. Abdominal computed tomography showed an isolated moderate-grade gastric distension, with smooth thin wall and no evidence of distal gastric, pyloric, or proximal duodenal obstructing masses (Figure 1). Afterward, the patient underwent esophagogastroduodenoscopy (EGD) which exhibited no proof of an obstructing lesion. Overall, the imaging studies excluded potential etiologies of mechanical obstruction and a diagnosis of liraglutide-induced gastroparesis was made.

Consequently, gastric suctioning through a nasogastric tube was carried out to treat her condition and the intake of liraglutide was ceased. The patient received a short course of antiemetics (ondansetron and metoclopramide) and her symptoms resolved completely. She was discharged home without complications after three days. Two weeks later, the patient was seen at the clinic, and she was doing well.

## Discussion

Gastroparesis is a syndrome that exhibits delayed gastrointestinal symptoms and slowed gastric emptying without a clinical or radiological proof of mechanical obstruction [2,5]. It is mostly encountered in young and middle-aged women, particularly those who are diabetics. Idiopathic etiologies are common and can account up to one-third of the cases [3,6]. Rare causes of gastroparesis may include connective tissue disorders and certain neurological diseases, such as Parkinson's disease [7].

In suspected gastroparesis cases, an endoscopy is generally performed to rule out gastric obstruction. In our patient’s case, gastroparesis-specific confirmatory tests-for example, four-hour solid-phase gastric emptying scintigraphy [8]-were not performed. This is because the patient lacked symptoms before liraglutide initiation and also her symptoms improved substantially after cessation of liraglutide.

The etiopathological factors of gastroparesis are not clearly delineated. However, acute variations in blood glucose levels, drug-related adverse effects, and abdominal pain transmitted through autonomic mechanisms are some potential causes [8]. Poor blood glucose control in patients with diabetes mellitus is a common underlying etiology of gastroparesis [9]. Hence, in our case report, it is possible that diabetes mellitus is a direct causative factor of gastroparesis. Nonetheless, the recent history of liraglutide intake largely favors liraglutide as the most likely contributing cause to the development of gastroparesis.

Liraglutide is a drug that belongs to a family of long-acting glucagon-like peptide one (GLP-1) receptor mimetics. It delays gastric emptying after three weeks of continuous treatment. Delayed gastric emptying decreases food intake as a fuller feeling is achieved, and thus liraglutide is often prescribed for weight loss [10]. The precise mechanism whereby liraglutide delays gastric emptying is not completely identified [11]. However, a large body of evidence supports the notion that GLP-1 receptor agonists, such as liraglutide, inhibit gastric emptying through parasympathetic (vagal) afferent-mediated central mechanisms [12].

Though gastroparesis is a documented adverse event of liraglutide, not much research has been conducted to verify its effect on patients. Rai and colleagues reported a case of liraglutide-induced acute gastroparesis in a 52-year-old gentleman with a history of well-controlled diabetes mellitus [13]. One study documented that three mg of liraglutide delayed gastric emptying at five and 16 weeks [10]. Patients exhibiting gastroparesis symptoms after taking liraglutide medication must be closely monitored. In our patient’s case, gastric suctioning and discontinuation of liraglutide had improved the symptoms, indicating that liraglutide might be the possible etiology of her acute presentation, given her symptoms’ temporal correlation to the recent commencement of liraglutide.

In our case, the patient was started on liraglutide to control her blood glucose as well as to promote weight loss [14]. In our case, we believe gastroparesis happened secondary to the acute high dose (three mg) of liraglutide. According to guidelines, our patient should have been started on 0.6 mg daily for the first week, and then the dose would be upscaled by 0.6 mg weekly until the maximum dose of three mg is reached [14].

Management of gastroparesis is largely symptomatic, including an increase in fluids intake as well as correcting nutritional deficiencies. Glycemic control is also of utmost importance and helps in regulating symptoms. Additionally, prokinetic medications can be used to curb nausea and vomiting [15]. Ceasing the use of the offending etiology, such as liraglutide use, is key to successful management.

## Conclusions

Gastroparesis is a syndrome of delayed gastric emptying without proof of obstruction. The present case demonstrates that symptomatic gastroparesis can be triggered by the initiation of liraglutide. Although uncommon, however, drug-induced gastroparesis should be contemplated in diabetic patients with a history of recent commencement of liraglutide, particularly at higher doses. Physicians need to follow careful prescribing practices to avoid side effects and severe cases associated with liraglutide. Symptomatic management of gastroparesis includes proper diet, fluid intake, prokinetic analogs, and discontinuation of etiological medicine.
